# GBP1 recruitment to actin-rich pedestals induced by extracellular Gram negative bacteria promotes pyroptosis

**DOI:** 10.1101/2025.09.25.678451

**Published:** 2025-09-27

**Authors:** Daniel J Bennison, Ishaan Chaudhary, Dharitri Chaudhuri, Justin Chun Ngai Wong, Priyanka Biswas, Qiyun Zhong, Wouter W Kallemeijn, Marianne Guenot, Arthur M Talman, Eva-Maria Frickel, Edward W Tate, Sandhya S Visweswariah, Gad Frankel, Avinash R Shenoy

**Affiliations:** 1 Department of Infectious Disease, Imperial College London, London, United Kingdom; 2 Department of Developmental Biology & Genetics, Indian Institute of Science, Bengaluru, India; 3 Department of Life Sciences, Imperial College London, London, United Kingdom; 4 The Francis Crick Institute, London, United Kingdom; 5 Department of Chemistry, Imperial College London, London, United Kingdom; 6 MIVEGEC, University of Montpellier, IRD, CNRS, Montpellier, France; 7 Department of Microbiology and Molecular Medicine, University of Geneva, Geneva, Switzerland

## Abstract

The IFNγ-induced GTPase guanylate binding protein 1 (GBP1) binds to lipopolysaccharide (LPS) on cytosolic Gram-negative bacteria and promotes pyroptosis via the recruitment and activation of caspase-4 on the bacterial outer membrane. Enteropathogenic and enterohaemorrhagic *Escherichia coli* (EPEC and EHEC, respectively) are extracellular pathogens that also induce LPS- and caspase-4-dependent pyroptosis. However, whether GBP1 is involved in this process remains unknown. EPEC and EHEC adhere intimately to intestinal epithelial cells via avid interactions between the bacterial adhesin Intimin and Tir (Translocated intimin receptor), a type 3 secretion system effector protein. Intimin-mediated clustering of Tir triggers actin polymerisation, leading to pedestal-like structures at bacterial attachment sites. Here we show that GBP1 is recruited to actin pedestals in human cells infected with EPEC and EHEC *in vitro* and mouse colonocytes infected with the EPEC-like murine pathogen *Citrobacter rodentium in vivo*. GBP1-dependent caspase-4 trafficking to these sites leads to pyroptosis and IL-18 release. To dissect the mechanism of GBP1 trafficking, we engineered a chimeric receptor (FcγR-Tir) by combining the intracellular signalling domain of Tir and the extracellular ligand-binding domain of the Fcγ receptor. Clustering of FcγR-Tir with IgG-coated beads produced ‘sterile’ actin-rich pedestals that were sufficient to recruit GBP1 independently of bacteria. Our findings reveal that cytosolic GBP1 is mobilised to sites of pathogen-induced actin remodelling independently of LPS. We establish that GBP1 not only operates as a pattern-recognition receptor but also orchestrates effector-triggered immunity against pathogens that hijack the actin cytoskeleton.

## Introduction

Guanylate Binding Protein 1 (GBP1) is the forerunner of a family of seven human IFNγ-inducible 65-75 kDa GTPases that promote antimicrobial responses and inflammasome activation in response to intracellular pathogens [1-5]. GBP expression can be induced by IFNγ in most cell types, reflecting their broad roles in cell-autonomous immunity [3]. GBPs were first implicated in pyroptosis of via mouse caspase-4 (also called caspase-11) in macrophages exposed to cytosolic LPS [6-8]. More recently, we and others have shown that human GBP1 drives pyroptosis by enabling the recruitment and activation of caspase-4 on the surface of cytosolic Gram-negative bacteria [9-12]. GBP1 directly binds LPS and forms polymeric ‘coats’ on the outer membrane of bacteria (e.g., *Shigella flexneri, Burkholderia thailandensis, Francisella novicida, Salmonella enterica* serovar Typhimurium, *Yersinia*) [10, 13-16]. GBP1 is proposed to act as a surfactant that extracts LPS and facilitates its binding to caspase-4, thereby stimulating its proteolytic activity [10, 17]. Active caspase-4 cleaves gasdermin D (GSDMD) leading to pyroptosis, and generates the bioactive form of IL-18, which promotes intestinal homeostasis and antimicrobial immunity [18]. GBP1 thus defends against invasive cytosolic Gram-negative bacteria by acting as a pattern-recognition receptor (PRR) for LPS to promote the pyroptotic removal of bacterial niches and release of immune cytokines.

Biochemically, the GTPase activity of GBP1, which drives GBP1 dimerisation and higher-order oligomerisation, is required for its recruitment to LPS [3-5]. In addition, GBP1 has a C-terminal farnesylation motif (^589^CTIS^592^) and a proximal polybasic region (^584^RRRK^587^), which contribute to GBP1 trafficking to cytosolic bacteria and pyroptotic responses [19, 20]. Other GBP family members, such as GBP2-5, may support pyroptosis, but their involvement is pathogen- and cell type-dependent [9-12]. In addition, GBP1 targets other intracellular bacteria (e.g., *Listeria*, *Mycobacterium*), protozoan parasites (e.g., *Toxoplasma gondii*) and viruses (e.g. HIV) [4], suggesting that GBP1 may detect signals other than LPS. The role of GBP1, if any, in detecting extracellular bacterial infection remains unexplored.

Enteropathogenic and enterohaemorrhagic *E. coli* (EPEC and EHEC, respectively) exemplify extracellular Gram-negative human pathogens that trigger caspase-4-dependent pyroptosis [21]. EPEC is among the leading causes of childhood mortality and morbidity in low- and middle-income countries [22], and EHEC causes fatal infection mainly in high-income countries [23]. These pathogens tightly attach to gut epithelia, manipulate the host actin cytoskeleton, and trigger the loss of brush border microvilli leading to characteristic attaching and effacing (A/E) lesions [24]. EPEC and EHEC remain on the outside of the host cell and deploy a type 3 secretion system (T3SS) to inject ~20-50 effectors to manipulate host signalling [25]. Clustering of the T3SS effector Tir on the host plasma membrane by the bacterial outer membrane adhesin Intimin triggers actin polymerisation leading to the formation of structures commonly referred to as bacterial ‘pedestals’ [26]. We previously showed that actin polymerisation by Tir^EPEC^ or Tir^EHEC^ drives rapid LPS- and caspase-4-dependent pyroptosis of human macrophages [27]. Moreover, during infection of IFNγ-primed non-phagocytic intestinal epithelial cells by EPEC, bacterial LPS can be internalised into cells in a Tir-dependent manner, leading to caspase-4 activation and pyroptosis [28, 29].

The Tir proteins of EPEC and EHEC share a hairpin topology and an Intimin-binding region within the extracytoplasmic loop. However, their C-terminal regions use distinct mechanisms to polymerise actin. Intimin-mediated clustering of Tir^EPEC^ results in its tyrosine phosphorylation (on Y474 in Tir^EPEC^) by Src tyrosine kinases, thereby generating a binding site for the adaptor proteins NCK1/2 [30, 31]. These adaptors recruit the actin nucleation-promoting factor NWASP (Neuronal Wiskott-Aldrich syndrome protein), which stimulates ARP2/3-dependent actin polymerisation [32]. In contrast, Tir^EHEC^ recruits NWASP-ARP2/3 via a second T3SS effector, the Tir-cytoskeleton coupling protein (TccP; also called *E. coli* secreted protein F-like from prophage U (EspFU)) [33, 34]. Tir^EHEC^ and TccP interaction is scaffolded by the host proteins IRTKS (Insulin receptor tyrosine kinase substrate) or IRSp53 (Insulin Receptor Substrate p53), which bind Tir^EHEC^ at its ^456^NPY^458^ motif [35-37]. *Citrobacter rodentium* (CR), an EPEC-like natural murine pathogen, is commonly used to model infection *in vivo* [38-40]. Tir mutants of CR unable to polymerise actin are less virulent *in vivo* during mouse infection, pointing to the crucial role of Tir in pathogenesis [41].

Here we investigated the role of GBP1 in pyroptosis in response to adherent Gram-negative bacteria in non-phagocytic epithelial cells. We reveal that GBP1 is recruited to actin-rich pedestals generated by Tir-Intimin signalling by EPEC and EHEC, and that GBP1 recruitment is independent of bacterial LPS. GBP1-dependent caspase-4 recruitment to pedestals is also independent of LPS, which is only required for stimulating caspase-4 activity. Thus, pathogen-induced actin polymerisation is a novel signal that mobilises cytosolic GBP1 for effector-triggered immunity (ETI) against extracellular pathogens.

## Results

### GBP1 promotes pyroptosis during EPEC and EHEC infection

**Figure 1.**
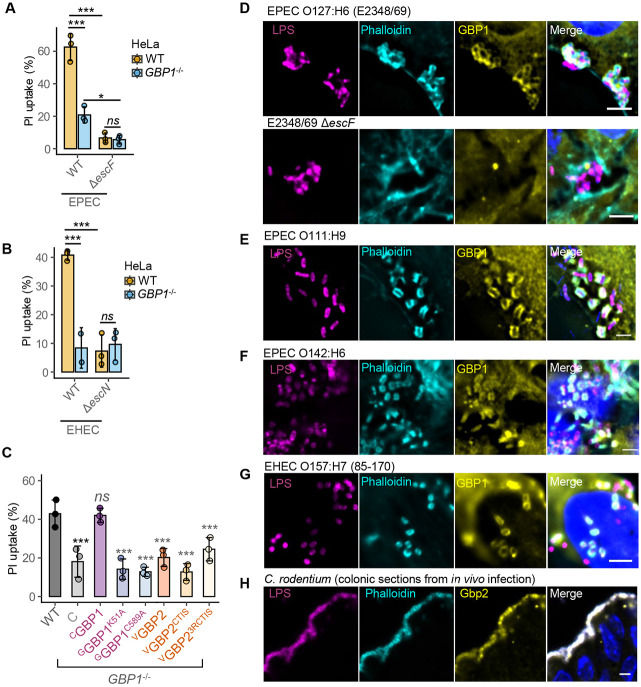
GBP1 promotes pyroptosis and is recruited to actin-rich pedestals of extracellular Gram-negative pathogens. (A) – (B) Percentage pyroptotic cell death as measured by propidium iodide dye uptake assays from IFNγ-primed wild-type (WT) or *GBP1*^−/−^ HeLa cells infected with wild-type (WT) EPEC O127:H6 (strain E2348/69) or an isogenic Δ*escF* mutant for 6 h (A) or WT EHEC O157:H7 (strain 85-170) or an isogenic Δ*escN* mutant for 8 h. Mean ± SD error bars with symbols representing data from n = 3 independent experiments are shown. * P < 0.05, *** P < 0.001, ns – not significant (P > 0.05) are two-tailed *P* values for the indicated comparisons from mixed effects ANOVAs. (C) Percentage pyroptotic cell death as measured by propidium iodide dye uptake assays from IFNγ-primed wild-type (WT) or *GBP1*^−/−^ HeLa cells stably expressing mCherry2 (C), mCherry2-tagged wild-type GBP1 or the indicated K51A or C589A mutants, or mVenus-GBP2 or the indicated mutants as indicated. Mean ± SD error bars with symbols representing data from n = 3 independent experiments are shown. *** P < 0.001, ns – not significant (P > 0.05) are two-tailed *P* values for comparisons between WT cells and cells expressing the indicated GBP1 or GBP2 variants from mixed effects ANOVAs. (D) Representative immunofluorescence images of IFNγ-primed HeLa cells infected with wild-type EPEC or Δ*escF* mutant for 2 h. Cells were stained with anti-LPS (to stain bacteria) and anti-GBP1 antibodies, phalloidin (to stain actin) and Hoechst (DNA dye) as labelled. Scale bar, 5 μm. (E) – (G) Representative immunofluorescence images of IFNγ-primed HeLa cells infected with EPEC O111:H9 (E), EPEC O142:H6 (F) or EHEC O157:H7 (strain 85-170) (G) as labelled. Infections were carried out for 3 h (E-F) or 5 h (G). Cells were stained with anti-LPS (to stain bacteria) and anti-GBP1 antibodies, phalloidin (to stain actin) and Hoechst (DNA dye) as labelled. Scale bar, 5 μm. (H) Representative immunofluorescence images of colonic sections from C57BL/6 mice infected with CR at 8-day post-infection. Sections were stained with anti-LPS (to stain bacteria) and anti-GBP1 antibodies, phalloidin (to stain actin) and Hoechst (DNA dye) as labelled. Data are representative of images from 5 infected mice. Data from the following number (n) of independent repeats: D, n = 6; E-F, n = 2; G, n = 5; H, n = 5.

We previously showed that EPEC O127:H6 (strain E2348/69) induces caspase-4-dependent pyroptosis in IFNγ-primed colonic epithelial cells [28, 29]. Similarly, in IFNγ-primed HeLa cells, wild-type (WT) EPEC induced pyroptosis as quantified by propidium iodide-uptake assays (Figure 1A) in a caspase-4–dependent manner (Figure S1A). In contrast, pyroptosis was not induced following infection by an isogenic T3SS-defective Δ*escF* mutant (Figure 1A). Deletion of GBP1 (*GBP1*^*−/−*^ HeLa cells) led to a marked reduction in pyroptotic lysis and IL-18 release upon EPEC infection (Figure 1A, S1B). Likewise, infection with wild-type EHEC O157:H7 (strain 85-170), but not an isogenic T3SS-defective Δ*escN* mutant, triggered pyroptosis in a GBP1-dependent manner (Figure 1B). Together, these results indicate that GBP1, caspase-4, and a functional bacterial T3SS are required for pyroptosis and IL-18 release during infection by EPEC and EHEC.

GBP1 prenylation and GTPase activity are essential for its pyroptotic functions during cytosolic bacterial infection [9-12]. We therefore used tetracycline (Tet)-inducible expression of mCherry2-GBP1 (^C^GBP1) variants to examine GBP1 activities required for EPEC-induced pyroptosis as described before [9, 42, 43]. Expression of ^C^GBP1, but not mCherry2 (C) alone, fully complemented the defect in pyroptosis and IL-18 production in *GBP1*^−/−^ cells (Figure 1C; S1C-E), attesting that the loss-of-function phenotype of these cells is specifically due to *GBP1,* and mCherry2-tagging does not affect protein function. Further, the expression of the GTPase-null K51A and prenylation-null C589A GBP1 variants failed to rescue pyroptosis or IL-18 release (Figure 1C; S1C-E), indicating that both properties are required for pyroptosis during infection by EPEC.

Overexpression of GBP2, which naturally undergoes geranylgeranylation and lacks a polybasic motif, can restore caspase-4-dependent pyroptosis in *Shigella flexneri-*infected *GBP1*^−/−^ epithelial cells, suggesting that GBP2 may compensate for the loss of GBP1 expression [44]. However, in contrast to *Shigella* infection, elevated expression of mVenus-GBP2 (^V^GBP2) in the *GBP1*^*−/−*^ cells did not restore pyroptosis or IL-18 release during EPEC infection (Figure 1C; S1C-E), indicating an essential role for GBP1 in this context. We also tested two chimeric variants of GBP2: one incorporating the GBP1 CTIS motif (leading to GBP2 farnesylation; ^V^GBP2^CTIS^) and another incorporating the CTIS motif plus the polybasic region of GBP1, yielding ^V^GBP2-^583^RRRKACTIS^591^ (^V^GBP2^R3CTIS^). Neither variant could restore pyroptosis or IL-18 secretion defects in *GBP1*^*−/−*^ cells upon EPEC infection (Figure 1C, S1C-E).

Altogether, GBP1 catalytic activity and prenylation are required for pyroptosis and IL-18 production in response to EPEC infection and these actions cannot be compensated by overexpression of GBP2 or with variants substituted with C-terminal regions of GBP1.

### GBP1 is recruited to actin-rich pedestals induced by extracellular bacteria

As GBP1 forms coats on the LPS of cytosolic bacteria, we used immunofluorescence microscopy to assess GBP1 localisation during infection by EPEC and related A/E pathogens. During infection, EPEC uses its bundle forming pilus (BFP) to form microcolonies on host cells [25]. Actin-rich pedestals were formed by 100 % microcolonies of WT EPEC at 2 h post-infection, and around 80 % of these were positive for endogenous GBP1 (Figure 1D, S2A), indicating extensive recruitment of GBP1 to EPEC attachment sites. In contrast, GBP1 did not traffic to attachment sites of the Δ*escF* strain, which forms microcolonies and attaches to similar extent as WT EPEC, but cannot inject effectors or elicit actin polymerisation (Figure 1D, S2A). CL40 human colonic epithelial cells exemplify well-differentiated primary-like cells with low epithelial to mesenchymal transition (EMT) signature, have an intact IFNγ response and do not carry mutations in genes involved in IFNγ-signalling or pyroptosis (e.g., *IFNGR, STAT1, CASP4, CASP5, GSDMD, NLRP3* or *GBP1-7*) as per the Colorectal Cancer Atlas [45-47]. Endogenous GBP1 in CL40 cells also colocalised with actin-rich pedestals of wild-type, but not Δ*escF*, EPEC (Figure S2B), indicating that GBP1 localisation to EPEC pedestals also occurs in physiologically relevant intestinal epithelial cells.

To rule out LPS O-antigen-specific recruitment of GBP1, we tested clinical EPEC isolates expressing distinct O-antigens. Actin-rich pedestals of EPEC O111:H9, O142:H6, and O119:H6, were also positive for GBP1 (Figure 1E-F, S2C). This indicates that GBP1 targets EPEC-induced actin-rich pedestals in epithelial cells in a strain- and O-antigen-independent manner. Likewise, GBP1 was recruited to actin-rich pedestals of EHEC O157:H7 (Figure 1G). Actin-rich pedestals were formed by ~65 % of EHEC bacteria at 5 h post-infection, with 100% of these pedestals also positive for GBP1 (Figure S2D), pointing to similarly ubiquitous targeting of EHEC pedestals. Having observed GBP1 recruitment to EPEC and EHEC pedestals *in vitro*, we asked whether GBP1 targets A/E pathogens *in vivo.* To address this, we stained sections from colons of C57BL/6 mice infected with CR at the peak of infection (8 days post-infection). We observed that Gbp2, the murine orthologue of human GBP1 [3-5], colocalised with the actin-rich pedestals of CR (Figure 1H).

GBP1 can be secreted from cells, including during bacterial infection [48]. It was therefore plausible that GBP1 can bind to EPEC extracellularly following its secretion. To test this, we performed immunofluorescence analyses with and without permeabilisation to limit anti-GBP1 antibody access to extracellular GBP1, if any. All EPEC expressing mVenus fluorescent protein were stained with anti-LPS antibodies with or without permeabilisation, confirming that EPEC remains outside cells during infection as expected (Figure S2E), and validating the experimental approach. No GBP1 colocalisation was observed with EPEC when cells were not permeabilised prior to immunostaining, revealing that GBP1 is intracellular and recruited to actin-rich structures underneath bacterial attachment sites (Figure S2E). Altogether, we conclude that cytosolic GBP1 traffics to actin-rich pedestals of extracellular, intimately adherent A/E pathogens *in vitro* and *in vivo*.

### GBP1 promotes caspase-4 recruitment to bacterial pedestals

**Figure 2.**
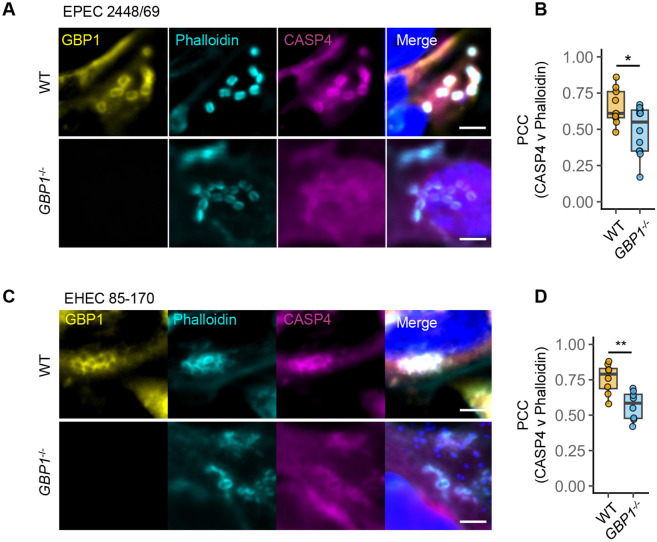
GBP1 enables caspase-4 recruitment to bacterial attachment sites. (A) Representative immunofluorescence images of IFNγ-primed wild-type (WT) or *GBP1*^−/−^ HeLa cells expressing YFP-Caspase4^C285S^ infected with wild-type EPEC for 2 h. Cells were stained with anti-GBP1 antibodies, phalloidin (to stain actin) and Hoechst (DNA dye). Scale bar, 5 μm. (B) Pearson’s correlation coefficient (PCC) for images from experiments in (A) assessing colocalization of caspase-4 and phalloidin are shown in wild-type (WT) or *GBP1*^−/−^ cells as labelled. (C) Representative immunofluorescence images of IFNγ-primed wild-type (WT) or *GBP1*^−/−^ HeLa cells expressing YFP-Caspase4^C285S^ infected with wild-type EHEC for 5 h. Cells were stained with anti-GBP1 antibodies, phalloidin (to stain actin) and Hoechst (DNA dye). Scale bar, 5 μm. (D) Pearson’s correlation coefficient (PCC) for images from experiments in (A) assessing colocalization of caspase-4 and phalloidin are shown in wild-type (WT) or *GBP1*^−/−^ cells as labelled. Data from n = 5 independent experiments, and each dot in B and D represent independent fields of view used for PCC measurements across experiments are shown. Box plots, median and interquartile range (IQR) are shown, with boxes indicating IQR, whiskers indicating 1.5x IQR and the line indicating the median. * P < 0.05, ** P < 0.01 are two-tailed *P* values for the indicated comparisons from independent samples Student’s *t* tests.

GBP1 facilitates caspase-4 recruitment to intracellular Gram-negative bacteria [9-12], therefore we tested caspase-4 localisation during A/E pathogen infection. Immunofluorescence microscopy revealed that endogenous caspase-4 localised to actin-rich pedestals of EPEC and EHEC (Figure S2F-G). As both caspase-4 and GBP1 antibodies that reliably detect their respective targets were raised in mice (Table S1), we exploited the YFP-Caspase-4^C258S^ reporter (a catalytically inactive variant that serves as a fluorescent tracer without causing cytotoxicity) that we previously developed to monitor caspase-4 recruitment to cytosolic *Salmonella* [9]. Like native caspase-4, YFP-caspase-4^C258S^ localised to > 95 % of GBP1-positive pedestals of EPEC and EHEC verifying that the YFP-caspase-4^C258S^ reporter mimics the localisation of the native protein (Figure 2A, 2C).

EPEC and EHEC adhered to similar extents and induced comparable actin pedestal formation in wild-type and *GBP1*^−/−^ cells, ruling out a role for GBP1 in bacterial attachment or pedestal formation (Figure 2A, 2C). We next queried whether caspase-4 localisation to pedestals is GBP1-dependent. Caspase-4 recruitment was noticeably reduced in *GBP1*^−/−^ cells, displaying diffused localisation throughout the cell (Figure 2A, 2C). In further support for GBP1-dependent recruitment of caspase-4, the Pearson’s correlation coefficient (PCC) for phalloidin (which stains polymerised actin) and caspase-4 at EPEC and EHEC pedestals in *GBP1*^−/−^ cells was significantly reduced as compared to wild-type cells (Figure 2B, 2D). Together, these results show that GBP1 is important for caspase-4 enrichment at sites of actin-polymerisation induced by these bacteria.

### GBP1 recruitment to pedestals requires Tir-driven actin polymerisation

**Figure 3.**
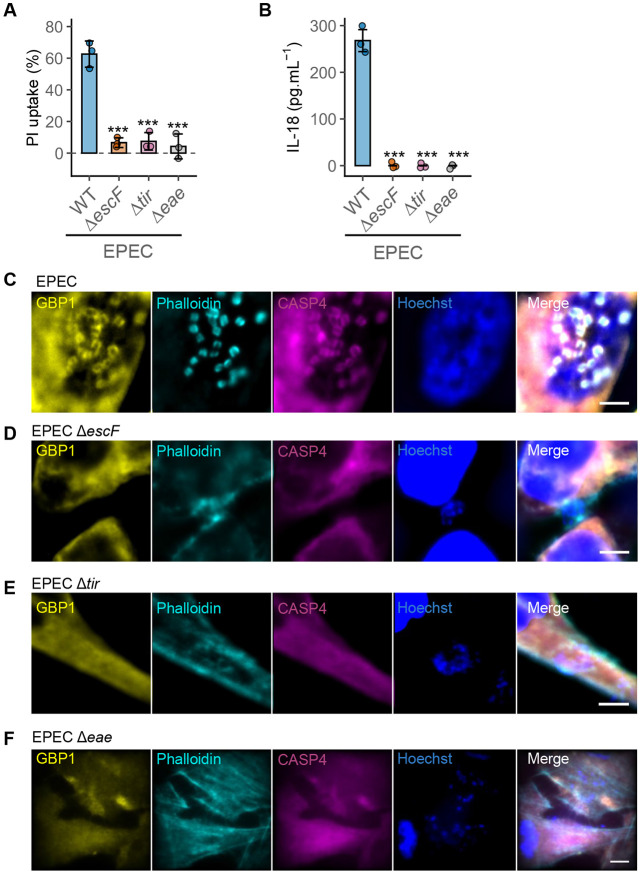
GBP1 recruitment and pyroptosis requires Tir-Intimin signalling leading to actin polymerisation. (A) – (B) Percentage pyroptotic cell death as measured by propidium iodide dye uptake assays (A) or IL-18 quantified by ELISA (B) from IFNγ-primed HeLa cells infected with the wild-type EPEC (WT) or the indicated mutants for 6 h. Mean ± SD error bars with symbols representing data from n = 3 independent experiments are shown. *** P < 0.001 are two-tailed *P* values for comparisons of mutant strains with WT from mixed effects ANOVAs. (C) – (G) Representative immunofluorescence images of IFNγ-primed HeLa cells expressing YFP-Caspase4^C285S^ infected with EPEC or the indicated mutants for 2 h. Cells were stained with anti-GBP1 antibodies, phalloidin (to stain actin) and Hoechst (DNA dye). Data from n = 3 independent experiments. Scale bar, 5 μm.

We next investigated the role of actin polymerisation in pyroptosis and the recruitment of GBP1-caspase-4 to A/E pathogen attachment sites. EPEC mutants that cannot form actin pedestals, such as the Δ*escF*, Δ*tir* and Δ*eae* (lacking the gene that encodes intimin; Figure S3A) strains failed to stimulate pyroptosis or IL-18 release (Figure 3A-B). Accordingly, neither GBP1 nor caspase-4 was recruited to the attachment sites of these EPEC mutants (Figure 3C-F). Comparable EHEC Δ*escN*, Δ*tir* and Δ*eae* mutants also did not trigger pyroptosis (Figure S3B), form pedestals, or recruit GBP1 or caspase-4 (Figure S3C-F). To establish whether Tir clustering, rather than ‘downstream’ actin polymerisation, is sufficient for GBP1 and caspase-4 recruitment, we used an EHEC Δ*tccP* mutant strain, which has intact Tir and Intimin proteins and can therefore lead to Tir clustering, but lacks the TccP protein to recruit NWASP-ARP2/3 for actin polymerisation (Figure S3A). As expected, EHEC Δ*tccP* bacteria did not form pedestals (Figure S3G), failed to induce pyroptosis (Figure S3B) and did not recruit GBP1 or caspase-4 (Figure S3G). These results underscore the requirement for EPEC- or EHEC-induced actin cytoskeleton manipulation via Tir-Intimin signalling for GBP1 mobilisation, which then promotes caspase-4 recruitment to these sites. Furthermore, the recruitment of both proteins correlates with pyroptosis and IL-18 release.

### GBP1 is recruited to ‘sterile’ actin polymerisation sites independently of LPS

**Figure 4.**
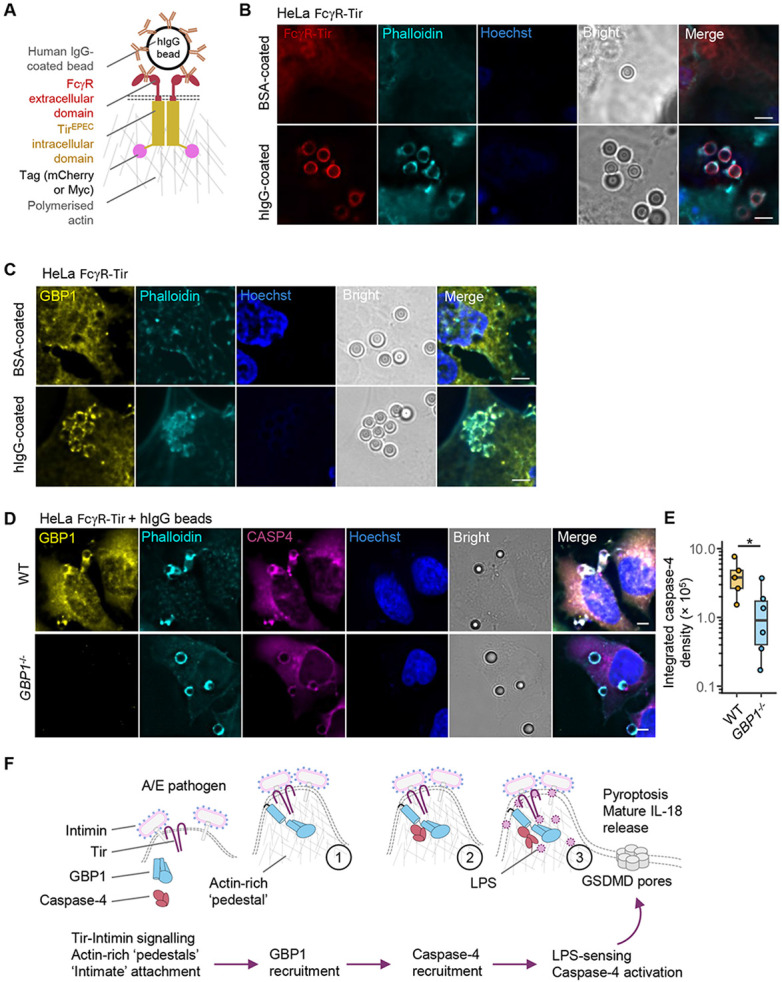
GBP1 is recruited to ‘sterile’ sites of actin polymerisation independently of LPS. (A) A schematic showing the reconstitution of Tir-driven actin polymerisation with an FcγR-Tir chimeric fusion protein containing the extracellular ligand-binding (IgG-binding) domain of FcγRIIA and the intracellular C-terminal region of Tir^EPEC^ that is sufficient for actin-polymerisation. Clustering of FcγR-Tir with sterile polystyrene beads (3.54 μm diameter) coated with human IgG (hIgG) results in actin-rich structures at sites of bead attachment. The FcγR-Tir chimaera with a C-terminal mCherry2 tag used for imaging in (B) or a non-fluorescent MYC tag in (C)-(D) behaved similarly (also see Figure S4). (B) Representative images from HeLa cells expressing FcγR-Tir (with C-terminal mCherry2 tag) treated for 3 h with sterile polystyrene beads coated with bovine serum albumin (BSA) or hIgG as labelled. Cells were stained with phalloidin (to stain actin) and Hoechst (DNA dye) and mCherry2 fluorescence showed FcγR-Tir localisation. Data are presentative of n = 3 independent experiments. Scale bar, 5 μm. (C) Representative images from HeLa cells expressing FcγR-Tir and YFP-Caspase-4^C258S^ treated for 3 h with sterile polystyrene beads coated with BSA or hIgG. Cells were stained with anti-GBP1 antibody, phalloidin (to stain actin) and Hoechst (DNA dye). Data are presentative of n = 3 independent experiments. Scale bar, 5 μm. (D) Representative images from wild-type (WT) or *GBP1*^−/−^ HeLa cells expressing FcγR-Tir and YFP-Caspase-4^C258S^ treated for 3 h with hIgG-coated sterile polystyrene beads. Cells were stained with anti-GBP1 antibody, phalloidin (to stain actin) and Hoechst (DNA dye). Data are presentative of n = 4 independent experiments. Scale bar, 5 μm. (E) Local caspase-4 intensity around the sites of actin-rich structures in WT or *GBP1*^−/−^ HeLa cells expressing the FcγTir fusion protein. Quantification of 5 (WT) or 6 (*GBP1*^−/−^) independent fields of view across at least 3 biological replicates was measured using ImageJ. Box plots, median and interquartile range (IQR) are shown, with boxes indicating IQR, whiskers indicating 1.5x IQR and the line indicating the median. * *P < 0.05* indicates two-tailed *P* value for the indicated comparisons from a Student’s *t* test. (F) Schematic summary of EPEC/EHEC-induced pyroptosis and IL-18 production in three-steps (indicated in numbers inside circles) and briefly outlined in the text blow. The initial attachment and microcolony formation (as in the case of EPEC) leads to the translocation of Tir and its clustering by Intimin expressed on the bacterial surface. In step 1, GBP1 is recruited by pathogen-induced actin polymerisation, in step 2 caspase-4 is recruited in a GBP1-dependent manner, and in Step 3, caspase-4 is activated by LPS at sites of focal bacterial attachment, leading to pyroptosis via gasdermin D (GSDMD) cleavage and IL-18 maturation.

We asked whether actin polymerisation driven by Tir can recruit GBP1 independently of bacterial LPS or other effectors. We note that the signal transduction by Tir^EPEC^ is analogous to eukaryotic immunoglobulin (Ig) Fragment crystallisable (Fc) receptors (FcR) because the crosslinking of the extracellular ligand-binding regions of both receptors triggers intracellular signalling via phospho-tyrosine motifs [49]. We therefore engineered a chimeric receptor composed of the signal peptide and the extracellular IgG-binding domain of the human Fcγ receptor IIA and the intracellular C-terminal region of Tir^EPEC^, which is sufficient for downstream signalling to NWASP-ARP2/3 [24] (i.e., FcγR-Tir fusion protein; Figure 4A). We reasoned that crosslinking of chimeric FcγR-Tir with human serum IgG (hIgG)-coated polystyrene beads would lead to clustering of the Tir intracellular domain leading to actin polymerisation (Figure 4A).

HeLa cells expressing FcγR-Tir exposed to hIgG-coated beads showed clustering of the FcγR-Tir chimaera and dense actin polymerisation around bead-attachment sites (Figure 4B). No FcγR-Tir clustering or actin polymerisation was observed with BSA-coated beads used as a negative control (Figure 4B). As an additional control, we developed FcγR-Tir^mut^ (with point mutations in the tyrosine residues in the intracellular domain of Tir that abolish actin polymerisation), which also failed to trigger actin polymerisation upon clustering with hIgG-coated beads (Figure S4A). Similarly, treatment with cytochalasin D, an actin polymerisation inhibitor, also blocked the formation of actin structures induced by hIgG-beads (Figure S4B). Together, this confirmed that signalling by FcγR-Tir is induced only upon clustering with antibody ligands leading to actin polymerisation through intracellular amino acid motifs known to be important for signalling in native Tir^EPEC^.

We next assessed endogenous GBP1 localisation in this experimental setting that produces ‘sterile’ actin-rich structures independently of bacteria or LPS. GBP1 was prominently recruited to actin-rich structures produced in FcγR-Tir cells treated with hIgG-beads but not BSA-beads (Figure 4C). GBP1 recruitment was blocked when actin polymerisation was abrogated with cytochalasin D treatment and in cells expressing FcγR-Tir^mut^ (Figure 4C, S4A-B). These experiments provided genetic and pharmacological evidence that actin polymerisation that mimics A/E pathogens is sufficient to elicit GBP1 mobilisation.

Similar to native bacterial infection, caspase-4 localised to sterile actin-rich structures produced in FcγR-Tir cells (Figure 4D), indicating that caspase-4 can also traffic to pedestals independently of LPS. Despite similar levels of actin-rich structures in *GBP1*^−/−^ FcγR-Tir cells treated with hIgG-beads, caspase-4 localisation at these structures was markedly reduced in *GBP1*^−/−^ cells as verified by quantification of caspase-4 intensity within the actin-rich structures around bead-attachment sites (Figure 4D-E). This implies that GBP1 is recruited to bacterial attachment sites independently of LPS and facilitates caspase-4 recruitment. As FcγR-Tir cells treated with BSA or hIgG beads for 6 h did not undergo appreciable levels of pyroptosis (Figure S4C), we inferred that GBP1-caspase-4 recruitment to actin-rich structures is not sufficient for triggering cell death, presumably due to the absence of LPS to stimulate caspase-4 proteolytic activity in this setting. We therefore conclude that GBP1-dependent and LPS-independent caspase-4 recruitment permits the detection of bacterial LPS at sites of focal bacterial attachment (Figure 4F).

## Discussion

Here we discovered that GBP1 responds to A/E bacteria-induced actin polymerisation and its effectiveness in surveillance of extracellular pathogen niches. GBP1 was recruited to ‘sterile’ actin polymerisation sites induced using our novel FcγR-Tir chimeric receptor, revealing that its mobilisation to bacterial attachment sites is LPS-independent. GBP1 also mediated caspase-4 recruitment in an LPS-independent manner. During A/E pathogen infection, GBP1-dependent caspase-4 recruitment leads to pyroptotic host-cell death and IL-18 release (Figure 4F). These findings add a new dimension to the antimicrobial actions of GBP1 which was previously characterised as a cytosolic PRR for LPS.

How LPS activates caspase-4/11 is of much interest given the highly inflammatory outcomes associated with pyroptosis [50, 51]. EPEC/EHEC and other Gram-negative bacteria shed LPS-containing outer membrane vesicles (OMVs) that can be internalised by host cells [52-54]. We previously showed that LPS enters IFNγ-primed human epithelial cells infected with EPEC in a manner that depends on Ca^2+^ influx through the TRPV2 ion channels, which is induced by Tir clustering by Intimin [28, 29]. Here we found that actin polymerisation by Tir recruits GBP1 to pedestals, which is followed by caspase-4 for LPS-sensing at bacterial attachment sites. We and others have previously showed that GBP1 and caspase-4 form a complex in response to bacterial infection or LPS transfection [9-12], therefore they may interact during EPEC/EHEC infection leading to their colocalization to bacterial pedestals. We propose that the pyroptosis induced by extracellular A/E pathogens proceeds via three steps: (i) trafficking of GBP1 to actin-rich pedestals to ‘prime’ the response, (ii) recruitment of caspase-4 for LPS-sensing, and (iii) stimulation of caspase-4 activity leading to pyroptosis and cytokine maturation (Figure 4F).

The loss of GBP1 prenylation and GTPase activity abolished its pyroptotic functions during EPEC infection, indicating that membrane recruitment and higher-order oligomerisation of GBP1 are essential for EPEC/EHEC-induced pyroptosis. We reason that GBP1 may operate like the Ras-family of small GTPases which also contain C-terminal prenylation signals and polybasic motifs that enable GTP-activity-dependent binding to phospholipids and membrane trafficking [55, 56]. GBP1 recruitment to bacterial attachment sites was strictly dependent on actin polymerisation but independent of the downstream signalling mechanism used by EPEC and EHEC (tyrosine phosphorylation by Tir^EPEC^ and TccP by Tir^EHEC^; Figure S3A). The signal(s) that induce GBP1 trafficking to Tir are not known. We ruled out the involvement of IRTKS/IRSp53 in recruiting GBP1, because both proteins are recruited to Tir during infection by the EHEC Δ*tccP* strain which did not recruit GBP1 [57]. We speculate that GBP1 recruitment may involve direct interactions with actin, which is a binding-partner of GBP1 [58], sensing of altered membrane curvature generated by I-BAR (Inverse – Bin1, amphiphysin, Rvs167) domain-containing proteins recruited by Tir [57, 59], phosphoinositides generated during Tir-signalling [60], or sugars, which have been proposed to recruit human GBP1 and mGbp2 to damaged vacuoles [61].

GBP1 mobilisation to EPEC/EHEC pedestals promoted pyroptosis, thus establishing its role in ETI against these pathogens. We also uncovered biochemical differences between the ETI role of GBP1 and previously reported features that support its LPS PRR activities. Here we observed GBP1 trafficking to actin-rich pedestals of pathogenic *E. coli* strains expressing distinct O-antigens; however, GBP1 trafficking to a rough LPS mutant of *S. flexneri* (that lack an O-antigen) is markedly reduced [19], suggesting that the O-antigen only influences the PRR activity of GBP1. More importantly, we established that GBP1 trafficking was completely LPS-independent as demonstrated by its recruitment to synthetic actin-rich structures generated using the FcγR-Tir chimeric receptor. In addition, the ETI role of GBP1 during EPEC infection could be not be sustained by GBP2 expression in *GBP1*^−/−^ cells, even though GBP2 can redundantly serve as an LPS PRR during *S. flexneri* infection of *GBP1*-deficient cells by aggregating LPS OMVs shed by the bacterium directly in the cytosol [44]. We posit that relatively low levels of LPS are internalised during infection by extracellular EPEC/EHEC, which requires GBP1-dependent actions due to its high affinity for LPS (dissociation constant ~60 nM [11]). Consistent with this, 100-times greater LPS concentration is required for its aggregation *in vitro* by GBP2 as compared to GBP1, pointing to the much weaker affinity of GBP2 for LPS [44].

It is likely that the high affinity of GBP1 for LPS is also the reason that its targeting to polymerised actin is context dependent. For instance, GBP1/mGbp2 do not target actin ‘comets’ generated by cytosolic *Shigella* and *Burkholderia* [19, 62-64]. In these scenarios, the abundant cytosolic LPS may be the dominant signal leading to the formation of coats directly on bacteria. Alternatively, because GBP1 is a farnesylated GTPase, it may preferentially target pathogen-induced cortical polymeric actin structures as opposed to cytosolic actin comets. Whether GBP1 is recruited to other pathogens that manipulate cortical actin, such as vaccinia virus, will be worth investigating in the future.

During CR infection of mice, caspase-4–dependent IL-18 production contributes to early neutrophil influx in the gut, which can be antagonised by the CR caspase-11/4 inhibitor, NleF [65]. The production of IFNγ by the host protects against CR and may help overcome the suppressive effect of NleF by upregulating mGbp2 expression [66, 67], which could be tested in *Gbp2-*deficient mice in the future. EPEC and EHEC have convergently evolved mechanisms for actin polymerisation, pointing to its important role in pathogenesis. Additionally, unlike the prototypical EPEC 2348/69, many typical and atypical strains of EPEC express TccP and/or the highly related TccP2 and deploy both actin polymerisation pathways, hinting at strong selection for actin polymerisation among A/E pathogens [68]. The remodelling of the actin cytoskeleton disrupts tight junctions and barrier function in the gut and promotes pathogenesis [39]. It is tempting to speculate that GBP1 has therefore evolved a mechanism to detect pathogen-induced actin polymerisation, which is the final result of EPEC/EHEC attachment, and trigger pyroptosis to clear away cells with adherent pathogens. This may provide a failsafe against EPEC/EHEC as their flagellins and T3SS systems evade detection by the NLRC4 inflammasomes which can readily detect similar molecules of *Salmonella* and *Shigella* [21, 25].

In summary, here we uncovered a novel interface between actin cytoskeleton manipulation and innate immunity via GBP1, i.e., LPS-independent GBP1-trafficking to sites of actin polymerisation induced by extracellular bacteria. The GBPs and caspase-4-like enzymes are conserved across vertebrate species, including domestic animals and pets that can be infected by A/E pathogens [69-72]. Whether GBPs similarly assist caspase-4-dependent responses in other animals deserves to be examined in the future. A plethora of pathogens or their secreted toxins that hijack the actin cytoskeleton [73, 74] may thus stimulate or evade GBP1-mediated ETI. Our discovery that GBP1 can respond to extracellular pathogens markedly broadens its scope as a sentinel of microbial infection.

## Materials & Methods

### Reagents, cell lines and bacterial strains

Key reagents, antibodies, cell lines and bacterial strains are listed in Table S1.

### Cell culture

Cell lines used in this study are listed in Table S1. Cells were maintained in a humidified incubator at 37 °C with 5 % CO_2_ and verified to be free from mycoplasma. HeLa and HEK293E cells were cultured in complete Dulbecco’s Modified Eagle’s Medium (DMEM) containing 4500 mg.L^−1^ glucose, 100 units (U).mL^−1^ penicillin, 100 μg.mL^−1^ streptomycin, 1 mM sodium pyruvate and 10 % (v/v) heat-inactivated foetal calf serum (HI-FCS). CL40 cells were grown in DMEM/F12 Ham (with 100 U.mL^−1^ penicillin, 100 μg.mL^−1^ streptomycin, 1 mM sodium pyruvate and 20 % (v/v) HI-FCS). Where required, cultures were supplemented with puromycin (2 μg.mL^−1^), blasticidin S (10 μg.mL^−1^) or zeocin (200 μg.mL^−1^). Prior to experiments, cells were primed with 10 ng.mL^−1^ IFNγ for 16 h, and where required, protein expression was induced by adding 200 ng.mL^−1^ doxycycline along with IFNγ.

### Growth of bacterial strains

All bacterial strains used in this study are listed in Table S1. Bacteria were routinely grown overnight in lysogeny broth (LB) at 37 °C with 180 rpm shaking. Appropriate antibiotics were added when necessary: ampicillin (100 μg.mL^−1^); kanamycin (30 μg.mL^−1^), chloramphenicol (20 μg.mL^−1^). Infections were carried out using DMEM-primed EPEC or EHEC, resulting in increased expression of LEE and non-LEE encoded virulence factors as described before [27-29]. For EPEC-priming before infections, overnight cultures grown in LB were diluted 1:50 into pre-equilibrated DMEM-low glucose (1000 mg.mL^−1^ glucose; Sigma D5546) supplemented with antibiotics as required, and incubated for 3 h in a humidified incubator at 37 °C with 5 % CO_2_. For EHEC-priming, cultures were grown in LB for 6 h and then in DMEM-low glucose for 18 h in a humidified incubator at 37 °C with 5 % CO_2_.

### Bacterial infections

DMEM-primed bacterial cultures were suspended in fresh DMEM at desired multiplicity of infection (MOI) following quantification by measuring optical density at 600 nm. Epithelial cell lines pre-treated with IFNγ were infected with bacteria at an MOI of 5 for microscopy or 30 for cell death assays (verified by counting CFU), unless otherwise stated. Infections were synchronised by centrifugation at 750 x*g* for 10 min. Typical EPEC form microcolonies during infection whereas EHEC does not and therefore takes longer to attach tightly to cells and translocate effectors, including Tir. EPEC infections were carried out for 2 h and EHEC infections for 5 h before the addition of gentamicin (200 μg.mL^−1^) to kill extracellular bacteria. For pyroptosis assays described below, EPEC infections were stopped at 6 h post-infection (hpi) and EHEC infections at 8 hpi. For microscopy, EPEC infection was stopped at 2 hpi and EHEC infection at 5 hpi, followed by fixation and processing as described below.

### Infection of mice with *Citrobacter rodentium* (CR)

All animal experiments complied with the Animals Scientific Procedures Act 1986 and U.K. Home Office guidelines and were approved by the local ethical review committee (PPL). Experiments were designed in agreement with the ARRIVE guidelines [75] for the reporting and execution of animal experiments, including sample randomization and blinding. Mouse experiments were conducted with five mice per group. Pathogen-free female 18-20 g C57BL/6 mice were purchased from Charles River Laboratories. All mice were housed in pathogen-free conditions, at 20-22°C, 30-40 % humidity on 12 h of light/dark cycle in high-efficiency particulate air (HEPA)-filtered cages with sterile corn cob bedding, nesting material, and enhancements (chewing toy, and opaque and transparent cylinders), and were fed with RM1 (E) rodent diet (SDS diet) and water ad libitum.

CR strain ICC169 (Nalidixic acid (Nal) resistant) [76] was grown overnight in LB supplemented with 50 μg.ml^−1^ nalidixic acid at 37 °C at 180 rpm, were centrifuged at 3000 x*g* for 10 min, and resuspended in sterile 1X phosphate buffered saline (PBS). Mice weight were recorded (d0) and they were infected with approximately 3 x 10^9^ CFUs in 200 μl sterile PBS by oral gavage as previously described [77]. For mock infection (uninfected mice), mice received 200 μl sterile PBS. The inoculum CFUs were confirmed by CFU quantification as previously described [77]. Infections were followed by plating mouse stools at 2-3 days post infection (dpi) onwards on LB agar (15 % v/v) + Nal plates as previously described [77].

### Immunohistostaining of colonic sections

Mouse colons were harvested at 8 dpi, fixed in 4 % paraformaldehyde (PFA) for 2.5 h, and immersed in 70 % ethanol. The fixed tissues were embedded in paraffin and sectioned at 5 μm. For immunofluorescence, sections were dewaxed by immersion in Histoclear solution twice for 10 min, followed by immersion in 100 % ethanol for 10 mins, twice, then 95 % ethanol for 3 min twice, 80 % ethanol for 3 min, and 1X PBS-0.1 % Tween, 20-0.1 % saponin (PBS-TS), for 3 min twice. The sections were heated for 30 min in demasking solution (0.3 % trisodium citrate, 0.05 % Tween-20 in distilled H2O). The slides were washed in PBS-TS, followed by blocking in PBS-TS supplemented with 10 % normal donkey serum (NDS) for 20 min. The slides were incubated overnight at 4 °C with mouse anti-CR polyclonal antibody (1:50), rabbit anti-GBP2 polyclonal antibody (1:100). The following day the slides were washed twice for 10 mins in PBS-TS, and incubated with the appropriate secondary antibody (1:100) and DAPI (1:1000) to stain DNA (Table S1). The slides were washed and mounted with ProLong Gold antifade mountant.

### Cell death assays and ELISAs

For propidium iodide (PI)-uptake assays, cells were seeded in 96-well black-wall clear-bottom plate at a seeding density of 2 x 10^4^ cells/well in 100 μL complete DMEM lacking antibiotics and phenol red (to reduce background fluorescence) [27]. Cells were primed for 16 h with 10 ng.mL^−1^ IFNγ or as specified in the figure legend. The medium was supplemented with 5 μg.mL^−1^ PI prior to infection and values in uninfected cells was used as baseline, which was subtracted from all values. Proportional uptake of PI was calculated as a percentage relative to the baseline-corrected values of uninfected cells given 0.05 % (v/v) Triton X-100. Infections were carried out at 37 °C with 5 % CO_2_ using a FLUOStar Omega microplate reader (BMG Labtech). Fluorescence measurements were taken at 540/10 excitation and 620/10 emission filters used.

For ELISAs, after infection, cells were centrifuged at 750 x*g* for 10 mins to collect supernatants. IL-18 was quantified using the Human Total IL-18 DuoSet ELISA kit (R&D Systems) according to the manufacturer protocol, using 96-well high-binding ELISA plates (Greiner). Samples were measured using a FLUOStar Omega microplate reader using absorbance at 450 nm, and corrected by subtracting absorbance at 540 nm.

### Generation of *GBP1-*knockout cells

HeLa cells seeded in 24-well plates at 1 x 10^5^ cells/well were co-transfected with 125 ng each of two plasmids encoding both constitutively expressed *Sp*Cas9 and *GBP1*-specific gRNA using TransIT-X2 Dynamic Delivery System (Mirus) as per the manufacturer guidelines, with transfection complexes prepared in Opti-MEM (Gibco). The gRNA sequences were as follows: GBP1 sgRNA1: CTCATAAGCTGGTACCACTC; GBP1 sgRNA2: TACATACAGCCAGGATGCAA. Together, these gRNAs aimed to remove the entire coding sequence of *GBP1*. Puromycin was added to the wells at a final concentration of 2 μg.mL^−1^ at 24 h post-transfection to select for transfected cells, and selection was continued for 72 h. Surviving cells were then trypsinised using 0.25 % trypsin-EDTA (Sigma), and limited dilutions were performed to a final concentration of 1 cell.mL^−1^ in complete DMEM to obtain isolated clonal islands upon plating in a 10 cm tissue-culture treated dish (Greiner). To confirm *GBP1* deficiency on the genetic level, the target locus was subject to Sanger sequencing, and GBP1 expression was assessed using western blotting.

### Stable silencing with miRNA30E

For stable knock-downs of target genes, 22-base oligonucleotides were cloned into the pMX-CMV-eYFP-miR30E backbone as described previously for caspase-4 [78, 79]. Briefly, 22 base oligonucleotides specific to the gene of interest were used in the enhanced miR30E version. The following sequences were used: non-targeting CTRL, TCACGACGTTGTAATACGACGT; *CASP4*, ATATCTTGTCATGGACAGTCGT. Using Sequence and Ligation Independent Cloning (SLIC) [80], these 22mer sequences were cloned into the retroviral pMXCMV-YFP-miR30E plasmid at the XhoI and EcoRI sites. pMXCMV-miR30E plasmids were then transduced into the recipient cell line as described below and expression of the gene of interest analysed via western blotting relative to the non-targeting control plasmid (Table S1). If required, eYFP-positive cells were selected via FACS using an ARIA III (BD Bioscience).

### Molecular biology methods for generating mutants and domain swaps

Throughout this study, SLIC was used for routine cloning, and Phusion polymerase or KOD hot-start polymerase was used for PCR for their high fidelity [80]. Oligonucleotides used are listed in Table S1, and all plasmid constructs were confirmed using Sanger sequencing (GeneWiz, Azenta). Expression of GBP1 was carried out using derivatives of the doxycycline-inducible template vector pTetDF-mCh in conjunction with the control vector pTet-rtTA-tTS [9]. Human FcγRIIA (aa 1-245) extracellular Fc-binding region and the Tir^EPEC^ (aa 387-550, either wild-type sequence or with Y454A and Y474A mutations to generate an actin polymerisation-defective FcγR-Tir^mut^) [81] and either mCherry2 or 2x MYC tag (GSEQKLISEEDLEQKLISEEDL) were fused by PCR and cloned into the retroviral packaging plasmid pMXCMV.

### Packaging and transduction of retro- and lenti-viral vectors

Virus-like particles were packaged and produced in HEK293E cells as described previously [9, 27, 78, 82, 83]. Briefly, retroviral (with pMX backbones) and second-generation lentiviral packaging (with pTet backbones), pCMV-MMLV-Gag-Pol and pHIV-1266 were used, respectively, in combination with the pseudotyping plasmid pCMV-VSV-G-Env. HEK293E cells were seeded in 24-well format at a density of 1 x 10^5^ cells/well in 1 mL complete DMEM supplemented with 10 mM HEPES pH 7.5, and transfected with a total of 1 μg plasmid DNA per well using Lipofectamine 2000 in a 1:2.5 ratio (w:v) relative to DNA. For retroviral packaging, a 5:4:1 ratio of plasmid-of-interest:Gag-Pol:VSV-G was used. For Lentiviral packaging, a 3:2:1 ratio of plasmid-of-interest:HIV-1266:VSV-G was used. Virus-containing supernatants harvested 48 h post transfection, HEPES (pH 7.5) was added to 10 mM final concentration (to stabilise the pH of cell supernatants), and filter sterilised through 0.45 μm low-binding filters (Pall Life Sciences). For transductions, 250-400 μL of virus-containing supernatant was added to recipient cells in 24-well format. At 48 h post-transduction, the appropriate selection antibiotic was added and cells maintained in it until stable pools were obtained. If required, antibiotic-selected pools were sorted via FACS using an ARIA III (BD Bioscience) for uniform target gene expression.

### Immunofluorescence staining, image acquisition and processing

Cells were plated on glass cover slips in 24-well format and infected as described above. At specified timepoints post-infection, cover slips were washed twice with serum-free DMEM, and fixed using freshly prepared PBS containing 4 % PFA for 20 min at room temperature (RT, ~20 °C). Cover slips were then washed twice with filter-sterilised PBS, and residual PFA was quenched using 50 mM NH4Cl prepared in PBS for 20 min at RT. Cover slips were washed twice with PBS, and, unless otherwise stated, permeabilised using Permeabilisation Buffer (PBS containing 0.3 % Triton X-100) for 3 min at RT, washed three times in PBS, and blocked in Blocking Buffer (PBS containing 5 % NDS) for 1 h before staining. Primary antibodies (Table S1) were diluted as per the manufacturer guidelines in Blocking Buffer + 0.1 % saponin and cover slips were inverted onto primary antibody solutions and incubated in a humidified chamber for 1 h at RT or 4 °C overnight. Cover slips were then washed three times in PBS in 24-well plates and inverted onto drops containing secondary antibodies (Table S1), phalloidin-Alexa Fluor-568 or phalloidin-Alexa Fluor-488 (Invitrogen), and Hoechst dye as appropriate in Blocking buffer for 1 h at RT in a humidified chamber. Cover slips were washed three times in filtered PBS, and mounted onto microscopy slides using ProLong Diamond Antifade Mountant (Fisher Scientific). Slides were allowed to cure for 24 h protected from light. In some experiments, imaging was performed in 96-well plates, and the same process was followed and samples imaged immediately following staining steps.

Images were acquired on a Zeiss CellDiscoverer 7 epifluorescence microscope fitted with an Axiocam 504 mono camera and a Colibri.2 light source. Samples were imaged at 100x magnification using a PApo 50x/1.2 water autoimmersion lens along with an afocal magnification lens with a range of 0.5-2x. The following LEDs were used: 385 / 470 / 520 / 567 / 590 / 625 nm along with Zeiss 90 HE, 91 HE and 92 HE filter sets. Images were processed using Zen Blue (Zeiss), and deconvolution and montages of pseudocoloured images were prepared using ImageJ (Fiji) software. Pearson’s Correlation Coefficient was calculated using the ImageJ plugin Coloc2 (Fiji) on regions of interest using original images. Integrated pixel density was measured using Fiji. Deconvolution used theoretical point spread functions (PSFs) matching image acquisition conditions and were generated using the Richards-Wolf method using PSF Generator ImageJ plugin. DeconvolutionLab2 ImageJ plugin with the Richardson-Lucy method with 10-25 iterations and appropriate PSFs were used for deconvolution.

### Triggering FcγR-Tir signalling with hIgG-coated beads

HeLa cells encoding FcγR-Tir fused to a C-terminal mCherry2 or MYC tag were seeded on glass coverslips in 24-well plates at a density of 8 x 10^4^ cells/well for 48 h. Cells were treated with 10 ng.mL-^1^ IFNγ for 16 h before treatment with beads. Polystyrene beads (3.54 μm diameter, Spherotech) were pelleted at 5,000 x*g* for 1 min, washed in 70 % ethanol and thrice in sterile PBS. For coating, beads were suspended in PBS containing either 50 μg.mL^−1^ of human IgG or 50 μg.mL^−1^ BSA and incubated for 18 h on a rotating wheel (10 rpm) at 4 °C. The beads were then washed three times in sterile PBS. Beads were counted and added to cells (at a density of 5 beads/cell) followed by centrifugation of the culture plate at 300 x*g* for 5 minutes. Incubation was carried out at 37°C with 5 % CO_2_ for 15 mins, followed by washing with serum-free DMEM, and replacing media with OptiMEM (supplemented with 1 mM sodium pyruvate). After 3 h, cells were washed twice with ice-cold PBS and fixed with 4 % PFA and samples prepared for immunofluorescence as described above. The integrated density of caspase-4 was calculated using ImageJ (Fiji) software by thresholding against actin-rich structures stained with phalloidin (MaxEntropy, Fiji), expanding a region of interest and quantifying total intensity for caspase-4 staining within the region.

### Data handling and statistics

For all quantitative experiments, a single mean was calculated from two or three technical replicates (i.e. replicate wells in a 96-well plate for PI-uptake assays). Experiments were repeated using distinct passages of cells on different days and independent cultures of bacteria to generate biologically and statistically independent data (represented by n in figure legends). Data were analysed using R (v.4.0 or higher, grafify package [84]) or GraphPad Prism (v.8.0 or higher) (Table S1). For bar charts, mean and standard deviation (SD) are displayed. For box plots, median and interquartile range (IQR) are indicated as defined in the figure legend, with boxes indicating IQR, whiskers indicating 1.5x IQR and the line indicating the median. Data plotting using grafify and ggplot2 [85] packages in R.

Statistical comparisons used mixed effects linear models fitted without or with random intercepts (experimental blocks) and analysed using the grafify R package. Residual distributions were analysed to ensure normal distribution when using parametric analyses, and log or logit transformations were used where necessary. Post-hoc comparisons used FDR adjustment (α = 0.05) used to correct *P* values for multiple comparisons.

## Supplementary Material

Supplement 1
