# Supplementary material for: GBP1 recruitment to actin-rich pedestals induced by extracellular Gram negative bacteria promotes pyroptosis
Source: bioRxiv. 2025 Sep 27:2025.09.25.678451. Preprint. [Version 1] doi: 10.1101/2025.09.25.678451 (PMC12485797; doi:10.1101/2025.09.25.678451)
Supplement: Supplement 1 [file media-1.pdf]

Supplementary Information

GBP1 recruitment to actin-rich pedestals induced by extracellular Gram-negative bacteria promotes pyroptosis

Daniel J Bennison[1], Ishaan Chaudhary[1], Dharitri Chaudhuri[1,2], Justin Chun Ngai Wong[1], Priyanka Biswas[3], Qiyun Zhong[1], Wouter W Kallemeijn[4,5], Marianne Guenot[3], Arthur M Talman[6], Eva-Maria Frickel[7], Edward W Tate[4, 5], Sandhya S Visweswariah[2,3], Gad Frankel[3], Avinash R Shenoy[1,8]

[1] Department of Infectious Disease, Imperial College London, London, United Kingdom
[2] Department of Developmental Biology & Genetics, Indian Institute of Science, Bengaluru, India
[3] Department of Life Sciences, Imperial College London, London, United Kingdom
[4] The Francis Crick Institute, London, United Kingdom
[5] Department of Chemistry, Imperial College London, London, United Kingdom
[6] MIVEGEC, University of Montpellier, IRD, CNRS, Montpellier, France
[7] Department of Microbiology and Molecular Medicine, University of Geneva, Geneva, Switzerland
[8] Correspondence: Room 4.40A, Flowers building, Armstrong Road, Imperial College, London SW7 2AZ
Email: a.shenoy@imperial.ac.uk

Supplementary Figures S1-S4
Supplementary Table S1

Bennison et al
Figure S1

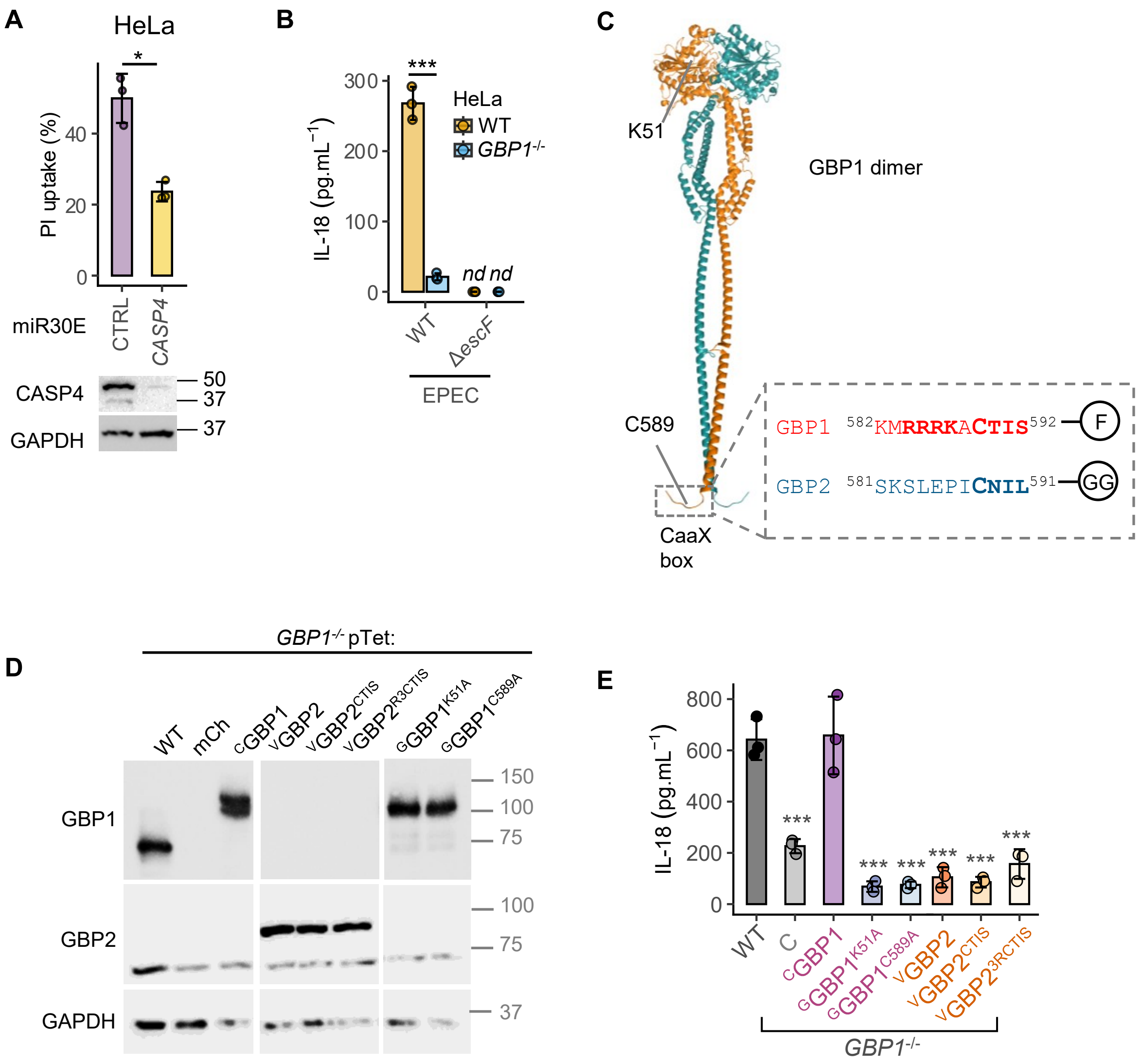

Figure S1. GBP1 is recruited to actin-rich pedestals of attaching and effacing (A/E) pathogens.

(A) Graph showing percentage pyroptotic cell death (top) and representative immunoblots (below) of HeLa cells stably expressing negative control (CTRL) or caspase-4 (*CASP4*)-targeting miR30E. Graph (top) shows percentage propidium iodide dye uptake assays in the indicated cells infected with EPEC for 6 h. Cells were primed with IFNγ (10 $ng.mL^{-1}$) ~16 h before infection. Mean ± SD error bars with symbols representing data from n = 3 independent experiments are shown. * $P < 0.05$ is a two-tailed *P* values for the indicated comparisons from mixed effects ANOVAs. Representative immunoblots (below) of Caspase4 and GAPDH (loading control). Data are representative of n = 3 independent experiments.

(B) ELISA quantification of IL-18 from supernatants of IFNγ-primed HeLa cells of the indicated genotypes infected with wild-type (WT) or Δ*escF* mutant of EPEC for 6 h. *** $P < 0.001$ is a two-tailed *P* values for the indicated comparisons from mixed effects ANOVAs.

(C) Ribbon structure of hGBP1 dimer (adapted from PDB: 8R1A), with individual monomers coloured orange and blue respectively. Key residues K51 and C589A are highlighted. C-terminal sequences for GBP1 and GBP2 are described with CaaX-box motifs and the GBP1 polybasic RRR motif shown in bold. The prenylation state of the protein is indicated with F or GG (farnesyl and geranylgeranyl, respectively).

(D) Representative immunoblots showing the expression of the indicated tetracycline-controlled GBP1 wild-type or the indicated variants in $GBP1^{-/-}$ cells. Cells were stably transduced with expression plasmids for the indicated proteins, treated with IFNγ (10 $ng.mL^{-1}$) and doxycycline (200 $ng.mL^{-1}$) for 16 h and cell lysates prepared for western blots. Data are representative of n = 2 independent experiments. Images shown are cropped from the same membrane at the same exposure to remove unnecessary lanes.

(E) IL-18 quantified by ELISA from IFNγ-primed wild-type (WT) or $GBP1^{-/-}$ HeLa cells stably expressing the GBP1 or GBP2 variants as labelled below and infected with wild-type EPEC for 6 h. Superscript C indicates mCherry2 or mCherry2-fusions, superscript V indicates mVenus fusion proteins and superscript G indicates GFP fusion proteins. Mean ± SD error bars with symbols representing data from n = 3 independent experiments are shown. * $P < 0.05$, *** $P < 0.001$, *ns – not significant* ($P > 0.05$) are two-tailed *P* values for the indicated comparisons from mixed effects ANOVAs.

Bennison et al
Figure S2

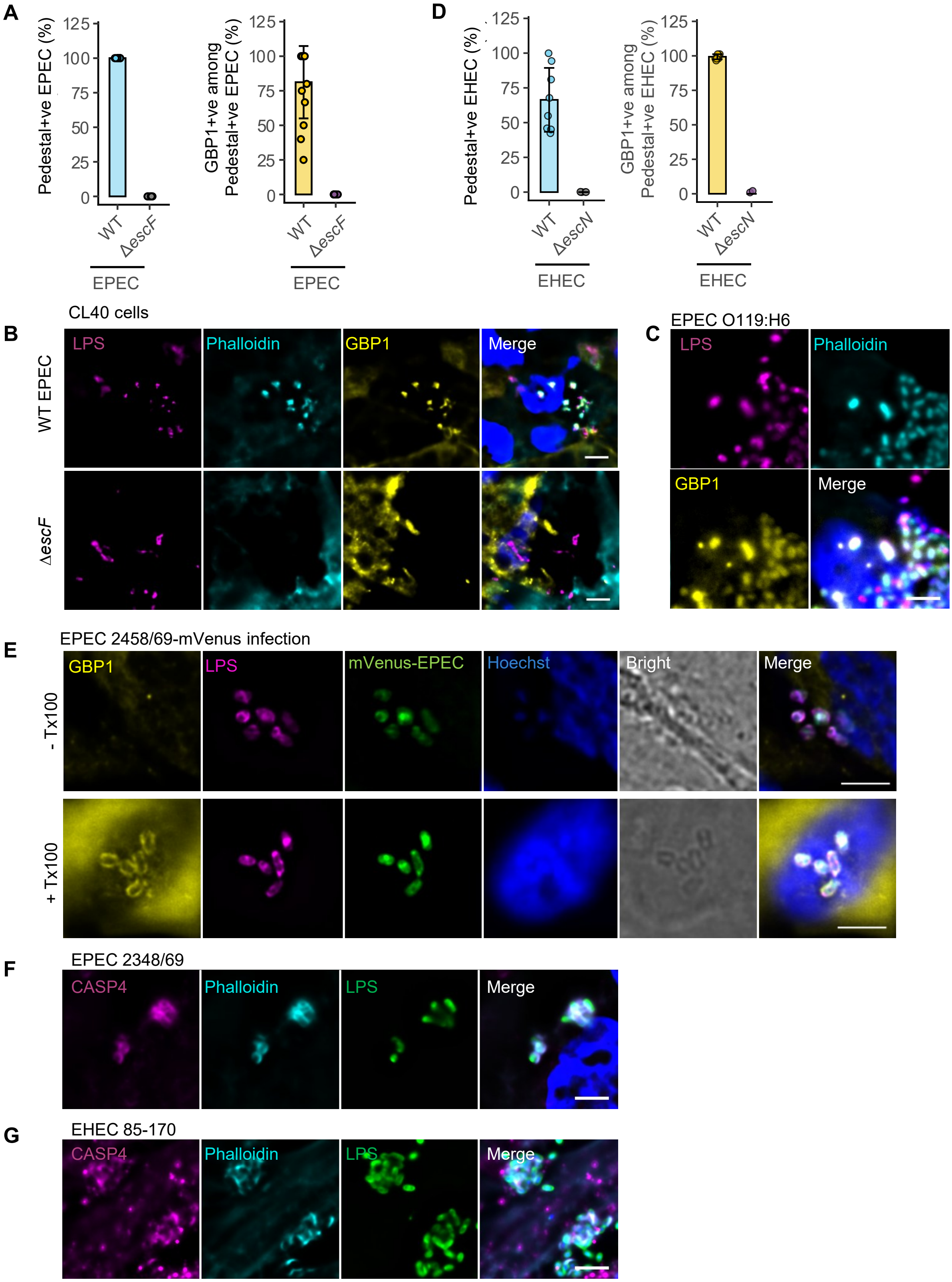

Figure S2. GBP1 is recruited to actin-rich attachment sites of extracellular EPEC.

(A) Quantification of microcolonies of wild-type (WT) or Δ*escF* mutant of EPEC infected in IFNγ-primed HeLa cells staining positive for actin-rich pedestals observed with phalloidin staining (Pedestal+ve) or positive for both phalloidin and GBP1 (GBP1+ve among Pedestal+ve) at 2 h post-infection (also see images in Figure 1). Mean ± SD error bars with symbols representing individual fields of view from n = 3 independent experiments are shown.

(B) Representative immunofluorescence images of IFNγ-primed human CL40 colonic epithelial cells infected with wild-type EPEC or Δ*escF* mutant for 1 h. Cells were stained with anti-LPS and anti-GBP1 antibodies, phalloidin-Alex568 (to stain actin) and Hoechst (DNA dye). Data are representative of n = 3 independent experiments. Scale bar, 5 μm.

(C) Representative immunofluorescence images of IFNγ-primed HeLa cells infected with EPEC O119:H6 for 3 h. Cells were stained with anti-GBP1 antibodies, phalloidin-Alex568 (to stain actin) and Hoechst (DNA dye). Data from n = 2 independent experiments. Scale bar, 5 μm.

(D) Quantification of wild-type (WT) or Δ*escN* mutant of EHEC infected in IFNγ-primed HeLa cells staining positive for actin-rich pedestals observed with phalloidin staining (Pedestal+ve) or positive for both phalloidin and GBP1 (GBP1+ve among Pedestal+ve) at 5 h post-infection (also see images in Figure 1). Mean ± SD error bars with symbols representing individual fields of view from n = 3 independent experiments are shown.

(E) Representative immunofluorescence images of IFNγ-primed HeLa cells infected with EPEC-mVenus for 1 h and then fixed and stained without and with permeabilization as labelled. Cells were fixed and either permeabilised for 3 min with 0.3 % Triton X100 or not prior to staining with anti-LPS and anti-GBP1 antibodies, and Hoechst (DNA dye). Data are representative of n = 3 independent experiments. Scale bar, 5 μm.

(F) – (G) Representative immunofluorescence images of IFNγ-primed HeLa cells infected with EPEC 2348/69 for 2 h (A) or EHEC 85-170 for 5 h (B) showing endogenous caspase-4 trafficking to actin-rich pedestals induced by bacteria. Cells were stained with anti-LPS (to stain bacteria) and anti-caspase-4 antibodies, phalloidin (to stain actin), and Hoechst (DNA dye). Data are representative of n = 2 independent experiments. Scale bar, 5 μm.

Bennison et al
Figure S3

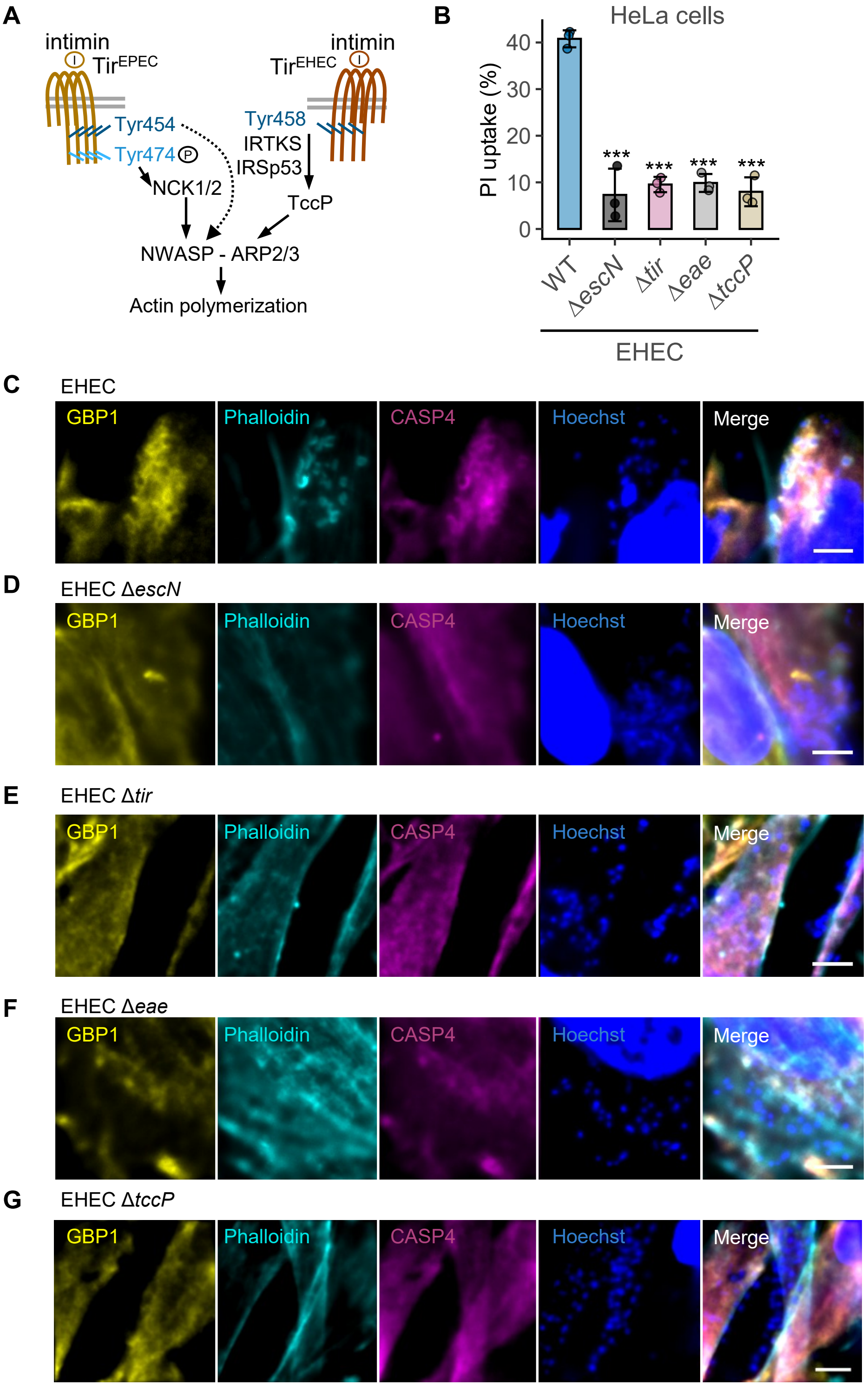

Figure S3. Tir-intimin signalling is essential for GBP1-caspase-4 recruitment to actin-rich pedestals of EHEC.

(A) A schematic showing the distinct pathways of actin polymerisation driven by Tir^{EPEC} and Tir^{EHEC}. TirEPEC mainly relies on Y474 tyrosine phosphorylation, NCK recruitment and NWASP-ARP2/3—dependent actin polymerisation. Tir^{EHEC} uses Y458 to recruit IRTKS/IRSp53 which bridge the interaction between Tir, TccP and NWASP-ARP2/3. The Y454 residue in Tir^{EPEC} can also recruit IRTKS/IRSp53, but this does not lead to actin-rich structures due to the lack of TccP in EPEC 2348/69 (shown by dotted arrow)

(B) Percentage pyroptotic cell death as measured by propidium iodide dye uptake assays of IFNg-primed HeLa cells infected with wild-type EHEC (WT) or the indicated mutants for 8 h. Mean ± SD error bars with symbols representing data from n = 3 independent experiments are shown. * $P < 0.05$, *** $P < 0.001$ are two-tailed *P* values for comparisons of mutant strains with WT from mixed effects ANOVAs.

(C) – (G) Representative immunofluorescence images of IFNγ-primed HeLa cells expressing YFP-$Caspase4^{C285S}$ infected with wild-type EHEC or the mutants for 5 h. Cells were stained with anti-GBP1 antibodies, phalloidin-Alex568 (to stain actin) and Hoechst (DNA dye). Data from n = 3 independent experiments. Scale bar, 5 μm.

Bennison et al
Figure S4

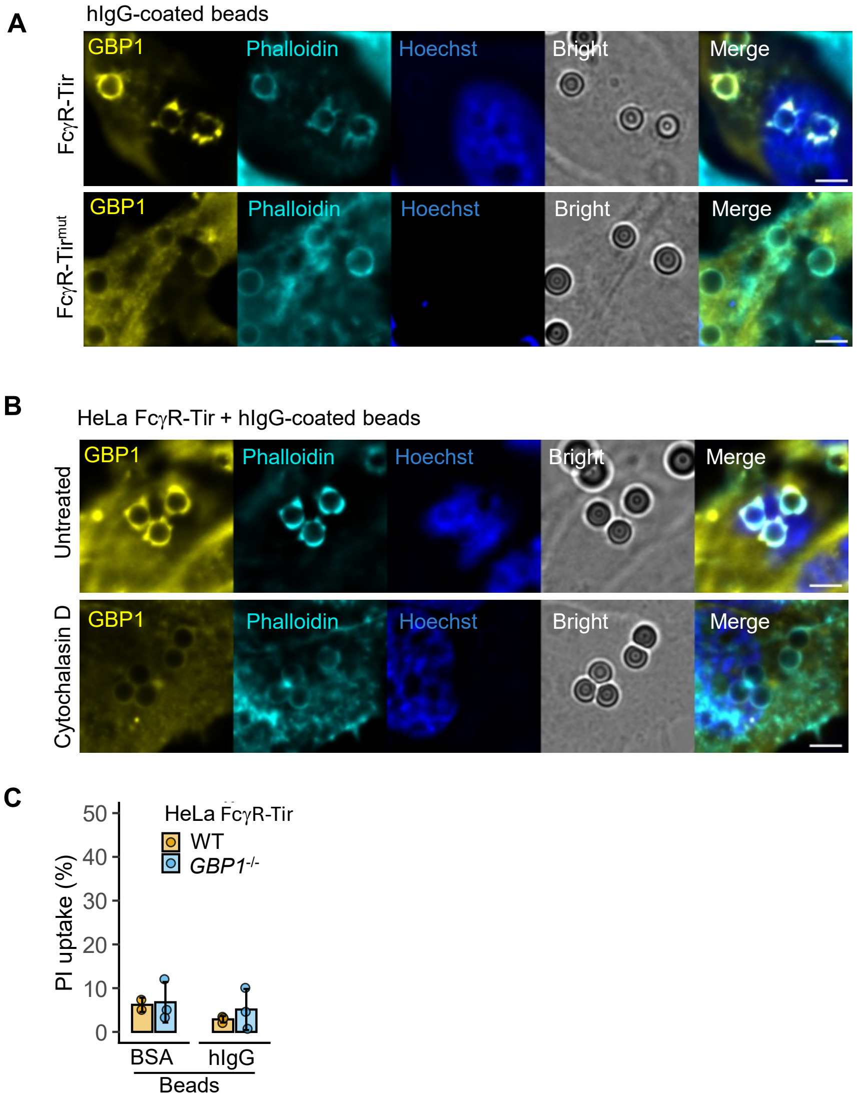

Figure S4. Inhibition of actin polymerisation by FcγR-Tir fusion protein blocks GBP1 recruitment to beads.

(A) Representative images from HeLa cells expressing FcγR-Tir or FcγR-Tir^{mut} (with C-terminal mCherry2 tag) treated for 3 h with sterile polystyrene beads coated with hIg. Cells were stained with an anti-GBP1 antibody, phalloidin (to stain actin) and Hoechst (DNA dye). Data are presentative of n = 3 independent experiments. Scale bar, 5 µm.

(B) Representative images from HeLa cells expressing FcγR-Tir (with C-terminal mCherry2 tag) treated for 3 h with sterile polystyrene beads coated with hIg in the absence or presence of the actin polymerisation inhibitor cytochalasin D (100 nM). Cells were stained with an anti-GBP1 antibody, phalloidin (to stain actin) and Hoechst (DNA dye). Data are presentative of n = 3 independent experiments. Scale bar, 5 µm.

(C) Percentage pyroptotic cell death as measured by propidium iodide dye uptake assays of IFNγ-primed HeLa cells of the indicated genotypes stably expressing FcγR-Tir cells treated for 6 h with BSA or hIgG-coated beads as indicated. Mean ± SD error bars with symbols representing data from n = 5 independent experiments are shown

Table S1. List of Cell lines, bacterial strains, reagents, kits and software

REAGENT or RESOURCE	SOURCE	IDENTIFIER
**Cell Lines**		
CL40	ATCC	
CL40 pMXCMV-miR30E-*CASP4*	This study	
CL40 pMXCMV-miR30E-*Ctrl*	This study	
HEK293E	[1-3]	
HeLa	ATCC	
HeLa *GBP1*$^{-/-}$	This study	
HeLa *GBP1*$^{-/-}$ pMX-FcγRIIa-Tir-MYC	This study	
HeLa *GBP1*$^{-/-}$ pTet-rtTA-tTS pTet-GFP-GBP1^{C589A}	This study	
HeLa *GBP1*$^{-/-}$ pTet-rtTA-tTS pTet-GFP-GBP1^{K51A}	This study	
HeLa *GBP1*$^{-/-}$ pTet-rtTA-tTS pTet-mCherry	This study	
HeLa *GBP1*$^{-/-}$ pTet-rtTA-tTS pTet-mCherry-GBP1	This study	
HeLa *GBP1*$^{-/-}$ pTet-rtTA-tTS pTet-mVenus-GBP2	This study	
HeLa *GBP1*$^{-/-}$ pTet-rtTA-tTS pTet-mVenus-GBP2^{CTIS}	This study	
HeLa *GBP1*$^{-/-}$ pTet-rtTA-tTS pTet-mVenus-GBP2^{R3CTIS}	This study	
HeLa pMXCMV-miR30E-*CASP4*	This study	
HeLa pMXCMV-miR30E-*Ctrl*	This study	
HeLa pMX-FcγRIIa-Tir-mCh	This study	
HeLa pMX-FcγRIIa-Tir-MYC	This study	
HeLa pMX-FcγRIIa-Tir^{YY454,474AA}-mCh	This study	
Human Colonic Organoids		
**Bacterial Strains**		
Citrobacter rodentium		ICC169
EPEC O111:H9	This study	
EPEC O142:H6	This study	
EPEC O111:H9	This study	
EHEC 85-170	[4]	O157:H7
EHEC 85-170 Δ*eae*		O157:H7
EHEC 85-170 Δ*escN*		O157:H7
EHEC 85-170 Δ*tccP*		O157:H7

EHEC 85-170 Δ*tir*		O157:H7
EPEC E2348/69		O127:H6
EPEC E2348/69 pTraCamBCD-mVenus	This study	O127:H6
EPEC E2348/69 TirY474A	[5]	O127:H6
EPEC E2348/69 Δ*eae*	[6]	O127:H6
EPEC E2348/69 ΔescF	[7]	O127:H6
EPEC E2348/69 Δ*tir*	[8]	O127:H6
EPEC-mVenus	This study	EPEC 2348/69 with a single copy of pTraCam-BCD-mVenus (Cm^R) at *att*Tn7 site downstream of *glmS*.
Stbl2 E. coli	[9]	
**Plasmids**		
pCMV-HIV-Gag-Pol	Walther Mothes [2]	
pCMV-MMLV 6 Walther Mothes	Walther Mothes [2]	
pCMV-VSV-G 6 Walther Mothes	Walther Mothes [2]	
pLentiCRISPR-GBP1-sgRNA#1 (sgRNA: CTCATAAGCTGGTACCAC TC)	This study	
pLentiCRISPR-GBP1-sgRNA#2 (sgRNA: TACATACAGCCAGGATGC AA)	This study	
pMXCMV-FcγRIIa-Tir-mCherry	This study	
pMXCMV-FcγRIIa-Tir-MYC	This study	
pMXCMV-FcγRIIa-$Tir^{YY454,474AA}$-mCherry	This study	
pMXCMV-YFP-$CASP4^{C258S}$	[2]	
pMXCMV-YFP-miR30E-CASP4		
pMXCMV-YFP-miR30E-Control		
pTet-GFP-$GBP1^{C589A}$	Fisch	
pTet-GFP-$GBP1^{K51A}$	Fisch	
pTet-mCherry	Fisch	
pTet-mCherry-GBP1	Fisch	
pTet-mVenus-GBP2	This study	
pTet-mVenus-$GBP2^{CTIS}$	This study	
pTet-mVenus-$GBP2^{R3CTIS}$	This study	
pTet-rtTA-tTS	[10]	
pTraCamBCD-mVenus-GlmSEPEC	This study	
**Primers**		
dGBP1_screen_seq_F	TCAATGCTGAAACTAGGTGGAA GT	This study - for screening whole GBP1 gene for knockouts

dGBP1_screen_seq_R	CCGTGTGCTAGTCCAAGCATT	This study - for screening whole GBP1 gene for knockouts
dGBP1_sgRNA1_seq_Rvs	TGAGGATACTTTGCTTATTTCGGTG	This study - for screening the sgRNA1 site
dGBP1_sgRNA2_seq_Fwd	ACAATTGCCACCACCACCAT	This study - for screening the sgRNA2 site
GCSFR-R_seq_Fwd	ACATTGCATACCCCACCCAG	This study - for screening most likely off-target effects of sgRNA1 and 2 to ensure no off-target effects
GCSFR-R_seq_Rvs	ATTGCTGCATCTCACCTGCT	This study - for screening most likely off-target effects of sgRNA1 and 2 to ensure no off-target effects
fam78b_seq_Fwd	TCGCGGTGTGAAGAACATGA	This study - for screening most likely off-target effects of sgRNA1 and 2 to ensure no off-target effects
fam78b_seq_Rvs	CACCCCTACAAACTGCCCTT	This study - for screening most likely off-target effects of sgRNA1 and 2 to ensure no off-target effects
YproteinPhosphatase_seq_Fwd	CCCTAGAATGATTTGGTGCCCT	This study - for screening most likely off-target effects of sgRNA1 and 2 to ensure no off-target effects
YproteinPhosphatase_seq_Rvs	TGATCCACCTCAAGCAGACAG	This study - for screening most likely off-target effects of sgRNA1 and 2 to ensure no off-target effects
GBP6_down_seq_fwd	ATCTGCCTCTTGATCTTTTAATCCT	This study - for screening most likely off-target effects of sgRNA1 and 2 to ensure no off-target effects
GBP6_up_seq_fwd	ACACTCAACTATGCTCACAACTC	This study - for screening most likely off-target effects of sgRNA1 and 2 to ensure no off-target effects
GBP2_F_pTetDFVenus	cgagctgtacaagGGATCCATGGCTCCAGAGATCAACTTGC	This study - for subcloning GBP2 variants into pTet-mVenus
GBP2CTIS_R_pTetDFVenus	CTTTCAAGACCTAGCTAGCGAATTCGATCGCTTAGCTTATGGTACATATTGGCTCCaatgatttgc	This study - for subcloning GBP2CTIS into pTet-mVenus
GBP2RRRKACTIS_R_pTetDFVenus	CTTTCAAGACCTAGCTAGCGAATTCGATCGCTTAGCTTATGGTACATGCCTTTCGTCGTCTtttgcttctcatctggatatccc	This study - for subcloning GBP2R3CTIS into pTet-mVenus
GBP2_pTetDF_mVenus_R	AGACCTAGCTAGCGAATTCGATCGCTTAGAGTATGTTACATATTGGCTCC	This study - for subcloning GBP2 into pTet-mVenus
Primer_FcgR_6aa-KZ-AfeI-Fwd	ccgtcagatccgctagcgctagccaccatgactatggagaCCCAAATGTCTCAGAATGT	Used to amplify the FcgR-EPEC Tir chimera adding a Kozak sequence and 6 amino-acid linker. SLICs with the AfeI site in the pMX-cmv plasmid
Primer_Tir_dStop-SLIC-Rev	AACGAAACGTACTGGTCC	Used to amplify the FcgR-Tir chimera removing the stop codon to enable tagging. SLICs with the mCherry or Myc Tag sequences.
Primer_mCherry_Tir_BamHI-Fwd	ggaccagtacgtttcgttggatccATGGTGAGCAAGGGCGAG	Amplifies mCherry to SLIC with the FcgR-Tir C-terminus, introducing a BamHI site

Primer_mCherry_pMX-EcoRI-Rev	ctcgaggcctgcaggaattcaCTTGTACAGCTCGTCCATG	Amplifies mCherry to SLIC with the FcgR-Tir and the EcoRI site within the pMX-cmv plasmid
Primer_Tir-Bam-Myc_pMX-Fwd	ggaccagtacgtttcgttGGATCCgagcagaagctgatctcagaggaggacctgGAACAAAAACTCATCTCAGA	Amplifies 2xMyc to SLIC with the FcgR-Tir C-terminus, introducing a BamHI site
Primer_Tir-Myc-pMX-EcoRI-Rev	CTCGAGGCCTGCAGGAATTCttacagatcctcttctgag	Amplifies 2xMyc to tag the FcgR-Tir and the EcoRI site within the pMX-cmv plasmid
Primer_FcgR_KZ_PacI_noCMV_Fwd	gccggatctagctagttaattaatgccaccATGACTATGGAGA	Used to amplify FcgRIIa to SLIC with pMX- removing the cmv promotor, and introducing a PacI site.
**Antibodies**		
Anti-hGBP1 mAb	ProteinTech	Cat# 67161-1-Ig<br>Clone 2A10E8<br>RRID: AB_2882457
Anti-hCASP4 mAb	MBL	Cat# M029-3<br>Clone 4B9<br>RRID: AB_590743
Anti-LPS pAb	Invitrogen	Cat# PA1-7244<br>RRID: AB_561201
Anti-Citrobacter pAb		
Anti-hGBP2 pAb	ProteinTech	Cat# 27299-1-AP<br>RRID: AB_2880836
Anti-hGBP5 mAb	CST	Cat# 67798<br>Clone D3A5O<br>RRID: AB_2799735
Anti-hGAPDH mAb	SCBT	Cat# sc-365062<br>Clone G9<br>RRID: AB_10847862
Donkey anti-Rabbit IgG (H+L) Cross-Adsorbed Secondary Antibody, HRP conjugate	Sigma	Cat# A16029<br>RRID: AB_2534703
Donkey anti-Mouse IgG (H+L) Cross-Adsorbed Secondary Antibody, HRP conjugate	Sigma	Cat# A16017<br>RRID: AB_2534691
Donkey anti-Mouse IgG (H+L) Highly Cross-Adsorbed Secondary Antibody, Alexa Fluor 488 conjugated	Invitrogen	Cat# A-21202<br>RRID: AB_141607
Donkey anti-Mouse IgG (H+L) Highly Cross-Adsorbed Secondary Antibody, Alexa Fluor 647 conjugated	Invitrogen	Cat# A-31571<br>RRID: AB_162542
Donkey anti-Rabbit IgG (H+L) Highly Cross-Adsorbed Secondary Antibody, Alexa Fluor 647 conjugated	Invitrogen	Cat# A-31573<br>RRID: AB_2536183

Donkey anti-Rabbit IgG (H+L) Highly Cross-Adsorbed Secondary Antibody, Rhodamine conjugated	Invitrogen	Cat# 31685<br>RRID: AB_429712
**Reagents**		
Acrylamide (40%) 37.5:1	Sigma Aldrich	Cat# A7168
Ammonium Chloride (NH4Cl)	Sigma Aldrich	Cat # 213330
Ampicillin	Sigma Aldrich	Cat# A9518
Blasticidin-HCl	InvivoGen	Cat # ant-bl-1
Bovine Serum Albumin	Sigma Aldrich	Cat # A3059
Chloramphenicol	Sigma Aldrich	Cat # C0378
Cytochalasin D	Sigma Aldrich	Cat# C8273
DAPI	Sigma Aldrich	Cat# D9542
Dimethyl Sulphoxide (DMSO)	Sigma Aldrich	Cat# D8418-50ML
DMEM/F12-HAM	Sigma Aldrich	Cat # D8062
DMEM/F12-HAM - Phenol Red Free	Life Technologies	Cat # 21041025
Doxycyline	Sigma Aldrich	Cat # 9891
Dulbecco's PBS	Sigma Aldrich	Cat # D8537
Dulbecco's Minimal Eagle's Media (DMEM) high glucose (4500 mg.L-1)	Sigma Aldrich	Cat # D5796
Dulbecco's Minimal Eagle's Media (DMEM) High Glucose (4500 mg.L-1) - Phenol Red Free	Invitrogen	Cat # 31053028
Dulbecco's Minimal Eagle's Media (DMEM) low glucose (1000 mg.L-1)	Sigma Aldrich	Cat # D6046
Foetal Bovine Serum - Heat Inactivated (low endotoxin)	Sigma Aldrich	Cat # F9665
Gentamicin	Sigma Aldrich	Cat # G1272
HEPES pH 7.2-7.5	Sigma Aldrich	Cat# H0887
hIgG, purified	Sigma Aldrich	Cat# I4506
Histoclear	Avantor	Cat # 101412-876
Hoechst 33342	ThermoFisher Scientific	Cat # H1399
Kanamycin	Sigma Aldrich	Cat# 60615
L-Glutamine	Sigma Aldrich	Cat # G7513
Lipofectamine™ 2000 Transfection Reagent	ThermoFisher Scientific	Cat # 11668030
Lysogeny Broth (LB)	Sigma Aldrich	Cat # L3022
Nalidixic Acid	MedChemExpress	Cat # HY-B0398
Non-Fat Dried Milk	VWR	Cat # A0830.1000
Normal Donkey Serum	Sigma Aldrich	Cat # D9663-10ML

NP-40	AppliChem	Cat # A1694
OptiMEM	Gibco	Cat # 31985062
Paraformaldehyde (PFA)	Sigma Aldrich	Cat # 128127
Penicillin/Streptomycin	Sigma Aldrich	Cat # P4333
Phalloidin-Alexa Flour 488	ThermoFisher Scientific	Cat # A12379
Phalloidin-Alexa Fluor 568	ThermoFisher Scientific	Cat # A12380
Phosphate Buffered Saline 10x	Fisher	Cat # 10214733
Pierce EDTA-free Protease Inhibitor Cocktail	ThermoFisher Scientific	Cat# A32955
PMSF	Sigma Aldrich	Cat # P7626
Polystyrene Beads	SpheroTech	Cat # PP-025-10
Precision Plus Protein Dual Color Standards	Bio-Rad	Cat # 1610374
Prolong Diamond Antifade	Invitrogen	Cat # P36961
Prolong Gold Antifade	Invitrogen	Cat # P36934
Propidium Iodide (PI)	Sigma Aldrich	Cat # P4170
Puromycin dihydrochloride from Streptomyces alboniger	Sigma Aldrich	Cat# P8833
PVDF membrane	Bio-Rad	Cat # 1620177
Recombinant hIFNγ	R&D Systems	Cat # 285-IF-100
Recombinant Streptavidin	BioLegend	Cat # 405150
Saponin	Sigma Aldrich	Cat # SAE0073
Sodium Dodecyl Sulphate	Sigma Aldrich	Cat # L3771
Sodium Pyruvate	Sigma Aldrich	Cat # S8636
SYBR Safe DNA gel stain	Life Technologies	Cat # S43102
Tetramethylethylenediamine (TEMED)	Sigma Aldrich	Cat # T9281
TransIT-X2 Dynamic Delivery System	Mirus	Cat # MIR 6004
Triton X-100	VWR	Cat # 9002-93-1
Trypan Blue	Sigma Aldrich	Cat # T8154
Trypsin-EDTA solution	Sigma Aldrich	Cat# T4049
Tween 20	Sigma Aldrich	Cat # P1379
Zeocin®	InvivoGen	Cat # ant-zn-1
**Commercial Kits**		
Clarity Western ECL Blotting substrate	Bio-Rad	Cat# 1705061
ECL Prime Western Blotting Detection Reagent	GE-Healthcare	Cat # RPN2236
Gel extraction kit	New England Biolabs	Cat # T1020
Human Total IL-18 DuoSet ELISA kit	R&D Systems	Cat # DY318-05

KOD Hot-Start Polymerase	Merck	Cat # 71086
Phusion High-Fidelity DNA Polymerase	New England Biolabs	Cat # M0530
PureLink Quick Plasmid Miniprep Kit	ThermoFisher Scientific	Cat # K210011
**Software**		
Fiji™	NIH	https://imagej.nih.gov/ij/
ggplot2 (v3.0 and above)	CRAN	https://cran.r-project.org/package=ggplot2
grafify (v4.0 and above)	CRAN	https://cran.r-project.org/package=grafify
GraphPad Prism 8.0	GraphPad Software	https://www.graphpad.com/scientific-software/prism/
Image Lab (v6.0.1)	Bio-Rad	http://www.bio-rad.com/en-uk/product/image-lab-software?ID=KRE6P5E8Z
MARS	BMG Labtech	https://www.bmglabtech.com/en/microplate-reader-software/
Microsoft Office 365 Enterprise	Microsoft	https://www.bmglabtech.com/en/microplate-reader-software/
Omega (v5.5)	BMG Labtech	https://www.bmglabtech.com/en/microplate-reader-software/
R (v4.0 and above)	The Comprehensive R Archive Network (CRAN)	https://cran.r-project.org/
Rstudio (Build 513)	Posit Software	https://posit.co/download/rstudio-desktop/
Zen Blue	Carl Zeiss	https://www.zeiss.com/microscopy/int/products/microscope-software/zen.html

References

1. Goddard, P.J., et al., *Enteropathogenic Escherichia coli Stimulates Effector-Driven Rapid Caspase-4 Activation in Human Macrophages.* Cell Rep, 2019. **27**(4): p. 1008-1017.e6.
2. Eldridge, M.J.G., et al., *The Atypical Ubiquitin E2 Conjugase UBE2L3 Is an Indirect Caspase-1 Target and Controls IL-1β Secretion by Inflammasomes.* Cell Rep, 2017. **18**(5): p. 1285-1297.
3. Mishra, V., et al., *IL-1β turnover by the UBE2L3 ubiquitin conjugating enzyme and HECT E3 ligases limits inflammation.* Nat Commun, 2023. **14**(1): p. 4385.
4. Levine, M.M., et al., *Escherichia coli strains that cause diarrhoea but do not produce heat-labile or heat-stable enterotoxins and are non-invasive.* Lancet, 1978. **1**(8074): p. 1119-22.
5. Wong, A.R., et al., *The enteropathogenic E. coli effector EspH promotes actin pedestal formation and elongation via WASP-interacting protein (WIP).* Cell Microbiol, 2012. **14**(7): p. 1051-70.
6. Marchès, O., et al., *EspJ of enteropathogenic and enterohaemorrhagic Escherichia coli inhibits opsono-phagocytosis.* Cell Microbiol, 2008. **10**(5): p. 1104-15.
7. Wilson, R.K., et al., *Role of EscF, a putative needle complex protein, in the type III protein translocation system of enteropathogenic Escherichia coli.* Cell Microbiol, 2001. **3**(11): p. 753-62.
8. Berger, C.N., et al., *The mechanisms used by enteropathogenic Escherichia coli to control filopodia dynamics.* Cell Microbiol, 2009. **11**(2): p. 309-22.
9. Trinh, T., et al., *STBL2: An Escherichia coli strain for the stable propagation of retroviral clones and direct repeat sequences.* 1994. **3**: p. 78-80.
10. Fisch, D., et al., *Human GBP1 is a microbe-specific gatekeeper of macrophage apoptosis and pyroptosis.* EMBO J, 2019. **38**(13): p. e100926.
